# Whole blood microRNA markers are associated with acute respiratory distress syndrome

**DOI:** 10.1186/s40635-017-0155-0

**Published:** 2017-08-30

**Authors:** Zhaozhong Zhu, Liming Liang, Ruyang Zhang, Yongyue Wei, Li Su, Paula Tejera, Yichen Guo, Zhaoxi Wang, Quan Lu, Andrea A. Baccarelli, Xi Zhu, Ednan K. Bajwa, B. Taylor Thompson, Guo-Ping Shi, David C. Christiani

**Affiliations:** 1 Department of Environmental Health, Harvard T.H. Chan School of Public Health, 665 Huntington Avenue, Boston, MA USA; 2 Department of Biostatistics, Harvard T.H. Chan School of Public Health, Boston, MA USA; 3 Department of Epidemiology, Harvard T.H. Chan School of Public Health, Boston, MA USA; 4 Department of Environmental Health, Department of Epidemiology and Biostatistics, Ministry of Education Key Laboratory for Modern Toxicology, School of Public Health, Nanjing Medical University, Nanjing, China; 5 Department of Critical Care Medicine, Peking University Third Hospital, Beijing, China; 6 Pulmonary and Critical Care Unit, Department of Medicine, Massachusetts General Hospital and Harvard Medical School, Boston, MA USA; 7 Department of Medicine, Brigham and Women’s Hospital and Harvard Medical School, Boston, MA USA

**Keywords:** ARDS, MicroRNA, LIPS, Whole blood

## Abstract

**Background:**

MicroRNAs (miRNAs) can play important roles in inflammation and infection, which are common manifestations of acute respiratory distress syndrome (ARDS). We assessed if whole blood miRNAs were potential diagnostic biomarkers for human ARDS.

**Methods:**

This nested case-control study (*N* = 530) examined a cohort of ARDS patients and critically ill at-risk controls. Whole blood miRNA profiles and logistic regression analyses identified miRNAs correlated with ARDS. Stratification analysis also assessed selected miRNA markers for their role in sepsis and pneumonia associated with ARDS. Receiver operating characteristic (ROC) analysis evaluated miRNA diagnostic performance, along with Lung Injury Prediction Score (LIPS).

**Results:**

Statistical analyses were performed on 294 miRNAs, selected from 754 miRNAs after quality control screening. Logistic regression identified 22 miRNAs from a 156-patient discovery cohort as potential risk or protective markers of ARDS. Three miRNAs—miR-181a, miR-92a, and miR-424—from the discovery cohort remained significantly associated with ARDS in a 373-patient independent validation cohort (FDR *q* < 0.05) and meta-analysis (*p* < 0.001). ROC analyses demonstrated a LIPS baseline area-under-the-curve (AUC) value of ARDS of 0.708 (95% CI 0.651–0.766). Addition of miR-181a, miR-92a, and miR-424 to LIPS increased baseline AUC to 0.723 (95% CI 0.667–0.778), with a relative integrated discrimination improvement of 2.40 (*p* = 0.005) and a category-free net reclassification index of 27.21% (*p* = 0.01).

**Conclusions:**

miR-181a and miR-92a are risk biomarkers for ARDS, whereas miR-424 is a protective biomarker. Addition of these miRNAs to LIPS can improve the risk estimate for ARDS.

**Electronic supplementary material:**

The online version of this article (10.1186/s40635-017-0155-0) contains supplementary material, which is available to authorized users.

## Background

Acute respiratory distress syndrome (ARDS) is a complex syndrome occurring in critically ill patients and is characterized as acute inflammation and infection caused by direct and indirect injury to the lung. ARDS affects approximately 200,000 people annually in the USA, carries a mortality rate of 40%, and is a major cause of intensive care unit (ICU) morbidity and mortality worldwide [1]. ARDS is mainly initiated by neutrophils accumulation and activation in the lungs, such cells can release inflammatory mediators and cytokines to damage pulmonary tissues [2, 3].

Importantly, the complex etiology and lack of reliable biomarkers have complicated ARDS diagnosis and treatment. Many protein-based biomarkers have been identified from plasma [4], but none have been translated to clinical diagnostic routines. More comprehensive study designs are needed to identify new mediators for ARDS pathogenic mechanisms [5]. Further, no previous studies have evaluated the potential or performance of whole blood microRNAs (miRNAs) to diagnose ARDS.

miRNAs, a group of small non-coding RNAs, regulate gene expression by binding to specific target sites on messenger RNA to either repress or degrade targets. Previous studies have discovered important roles for miRNAs in many disorders, including inflammation and infection [6, 7]. Thus, miRNA expression patterns may be able to serve as diagnostic biomarkers for better disease detection [8, 9]. Studies have suggested miRNAs are involved in ARDS development. In a rat model of ARDS, miRNA profiling of lung tissue demonstrated altered expression of multiple miRNAs compared to control tissues [10]. We recently demonstrated that whole blood miRNAs can be potentially valuable for predicting ARDS 28-day mortality due to their related function in multiple organ failure (MOF), which is a primary risk factor of ARDS patient mortality. Indeed, a miRNA panel is comparable to APACHE III in mortality prediction ability [11].

Yet, to date, no study has tested whether whole blood miRNAs may serve as biomarkers for ARDS risk. Whole blood offers advantages for miRNA profiling compared to other tissue types because it contains rich immune cell- and tissue-specific miRNAs with low risk of noise from additional serum or plasma isolation steps or sample contamination [12]. Therefore, we compared miRNA expression in whole blood preparations from two large populations of ARDS patients and critically ill at-risk controls.

## Methods

### Study population and design

This nested case-control study was part of an ongoing Molecular Epidemiology Study of ARDS (MEARDS) at Harvard Medical School that was initiated in 2000. MEARDS has more than 4000 patients and includes both ARDS patients and at-risk controls who were critically ill patients admitted to the ICU of Massachusetts General Hospital (MGH; Boston, MA) or Beth Israel Deaconess Medical Center (BIDMC; Boston, MA) [13, 14]. Commonly known ARDS risk factors and their definitions are listed in Additional file 1: Table S1. Detailed inclusion criteria are described in the “Supplemental Methods and Results” section in Additional file 1 and illustrated in Additional file 1: Figure S1. All ARDS subjects met the Berlin diagnostic definition [15]: timing of ARDS was within 1 week of a known clinical insult or new or worsening respiratory symptoms; chest imaging showed bilateral opacities (not fully explained by effusions, lobar/lung collapse, or nodules); respiratory failure was not fully explained by cardiac failure or fluid overload; and ARDS severity was based on PaO_2_/FiO_2_ ratio. Exclusion criteria included ages younger than 18 years old, diffused alveolar hemorrhage or chronic lung disease, which may mimic ARDS, and directive to withhold intubation. Patients with neutropenia not secondary to sepsis and immunosuppression secondary to medication or diseases such as HIV infection were excluded. Treatment with granulocyte colony-stimulating factor or inhibitors of tumor necrosis factor was also excluded [13]. Patients were enrolled in the study immediately after meeting all inclusion criteria [15]. Institutional review boards of MGH, BIDMC, and Harvard T.H. Chan School of Public Health approved this study.

**Fig. 1:**
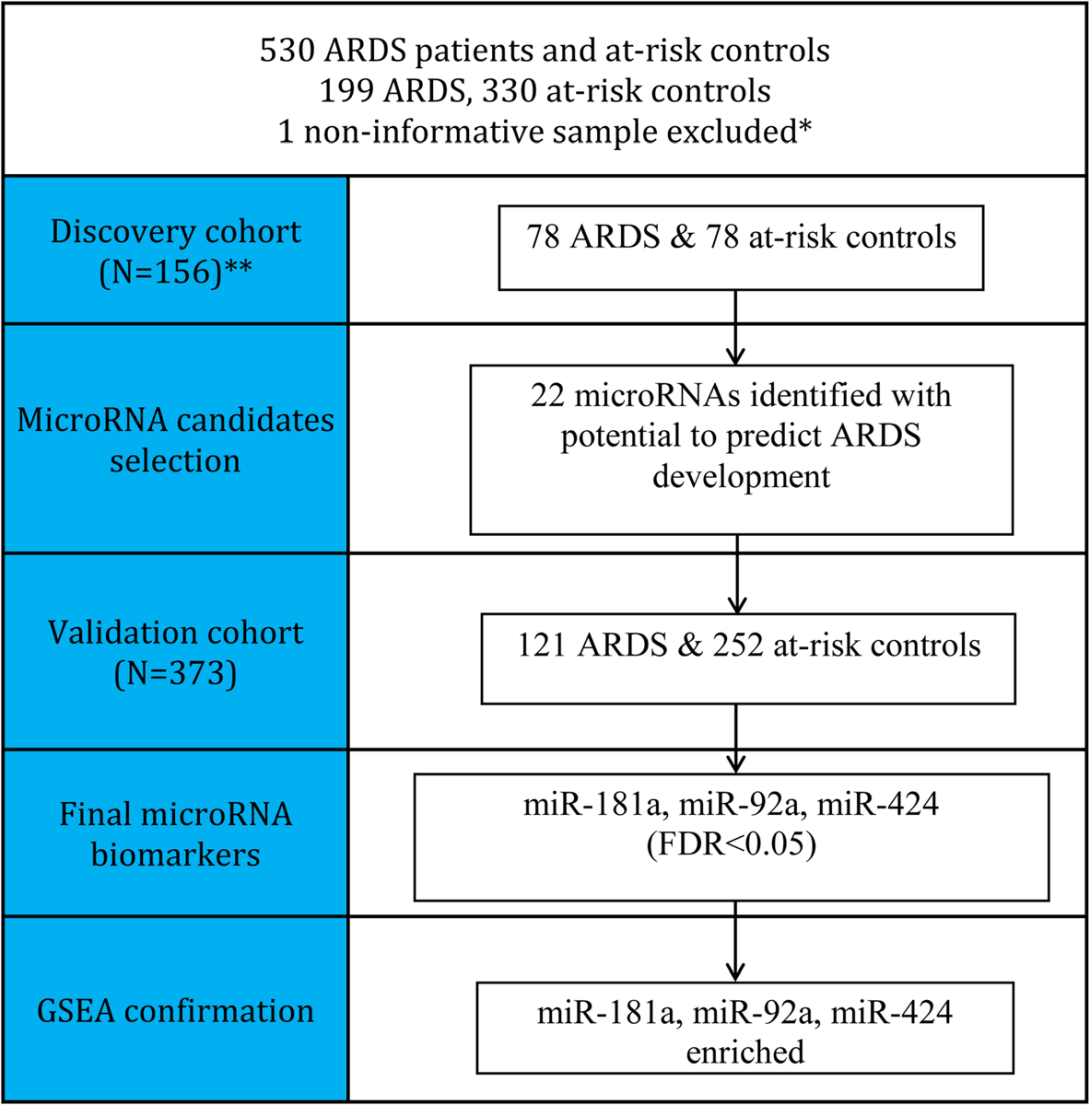
Study design of discovery cohort, validation cohort, and gene set enrichment analysis (GSEA). *One control sample was excluded due to few detectable miRNAs. **Discovery cohort ARDS and at-risk controls were matched by age (± 5 years) and gender

We used a two-phase study with a total of 530 participants (recruited during 2003–2012), including 199 ARDS and 330 at-risk controls. One patient without information was excluded (Fig. 1). The discovery population (*n* = 156) included 78 ARDS patients (cases) and 78 at-risk patients (controls), matched by age (± 5 years) and sex. Twenty-two miRNAs were used to build an ARDS risk factor panel that included one independent validation cohort (*n* = 373) containing 121 ARDS cases and 252 controls.

### RNA isolation

Peripheral whole blood from 530 participants was collected in Tri Reagent solution (Molecular Research Center, Cincinnati, OH) within 24 h of participant enrollment and stored at − 80 °C. Tri Reagent is a robust miRNA stabilization method for long-term storage and can generate reproducible results without degradation [16]. Total RNA containing small RNA was extracted from whole blood. RNA quality was assessed on an Agilent 2100 Bioanalyzer (Agilent Technologies, Palo Alto, CA), and RNA integrity numbers (RIN) were reported. Total RNA with RIN of 6.5–10 was processed for complementary DNA synthesis using TaqMan Megaplex RT primer pools A or B and then amplified with Megaplex PreAmp primers (Applied Biosystems, Foster City, CA). One control sample in the validation cohort was excluded due to few detectable miRNAs.

### miRNA profiling

In the discovery phase, we used the TaqMan OpenArray Human MicroRNA Panel (Applied Biosystems) according to the manufacturer’s instructions and detected expression of 754 human miRNA transcripts. After quality control screening, we selected 294 miRNAs for discovery cohort analysis. Twenty-two miRNAs were selected from the discovery cohort for further validation in an independent validation cohort. In the validation cohort, we used OpenArray QuantStudio system to customize miRNA expression arrays (Applied Biosystems). Technical consistency of assays within and across cohorts was also tested using three random samples.

### Statistical analysis

We applied stringent quality control (QC) criteria for miRNA analysis in order to maintain the confidence of statistical results: good amplification score > 1.1, Cq confidence > 0.8, high expression (Ct < 30), and detectable expression in more than half of the samples. We performed both univariate and multivariate logistic regression to identify miRNA candidates that were associated with ARDS status. Odds ratios (OR) and 95% confidence intervals (CI) were calculated. Discovery miRNA candidate selection was based on the following criterion: fold change > 1.5 or < 0.67 from the logistic regression model in at least one of four normalization methods [9]. Gene set enrichment analysis (GSEA) was used to investigate if candidate miRNAs were significantly enriched in the whole miRNA gene set [17].

To build the miRNA risk factor model, we used miRNAs that were validated in all cohorts with the same effect directions, and we computed sensitivity, specificity, accuracy, and area-under-the-receiver operating characteristic-curve (AUC) to assess performance of risk factors. To compare miRNA risk factors, we considered the base model for ARDS risk factor assessment to include only Lung Injury Prediction Score (LIPS) [18]. Based on our miRNA results, we included miR-181a, miR-92a, and miR-424, in combination with the LIPS model, in a miRNA classifier for ARDS risk factor evaluation. To further assess the incremental diagnostic power of those three miRNAs when added to the baseline LIPS risk model, we computed integrated discrimination improvement (IDI) and net reclassification index (NRI) (“Supplemental Methods and Results” section in Additional file 1), which offer an intuitive way of quantifying improvement offered by new biomarkers [19].

Values of *p* < 0.05 or false discovery rate (FDR) *q* < 0.05 were considered significant. All analyses were performed with R software (v.3.3.0) and Statistical Analysis System software (v.9.3, SAS Institute).

## Results

Demographic features and clinical variables of the discovery and validation cohorts are presented in Additional file 1: Table S2. In all experiments, we distributed samples such that age, sex, case-control status, and RNA quality were balanced with respect to the day of purification and the day of analysis or plate number and were randomized within each day and plate. This aspect is important to reduce confounding effects from technical variation, such as plate-to-plate variation and purification differences [20].

### miRNA screening and validation

In total, 754 miRNA transcripts were identified from the discovery cohort. We only included 294 miRNAs that met our QC inclusion criteria to maintain robust statistical results. Ultimately, 22 miRNAs were selected based on fold change as candidate risk factors for ARDS from the logistic regression model (Table 1). Of the 22 miRNAs, 3 miRNAs—miR-181a, miR-92a, and miR-424—remained significant risk factors (OR > 1.0) or protective factors (OR < 1.0) for ARDS in the validation cohort after multiple testing adjustment by FDR (Benjamini–Hochberg) (Table 1). From fixed effect meta-analysis, 14 miRNAs appeared to be significant risk/protective factors for ARDS. Of those 14 miRNAs, miR-181a, miR-92a, and miR-424 demonstrated the strongest associations with ARDS risk (*p* < 0.001) (Table 1).

**Table 1:** miRNA associations with ARDS in discovery cohort, validation cohorts, combined cohorts, and meta-analysis

	Discovery cohort (*n* = 156)	Validation cohort (*n* = 373)		Meta-analysis (*n* = 529)	
MicroRNA	OR (95% CI)	OR (95% CI)	FDR *q*	OR (95% CI)	*p*
miR-424	0.52 (0.29–0.93)	0.78 (0.66–0.94)	0.022	0.77 (0.67–0.9)	< 0.001
miR-92a	1.60 (1.11–2.31)	1.75 (1.26–2.43)	0.022	1.69 (1.32–2.17)	< 0.001
miR-181a	1.75 (1.03–2.97)	1.76 (1.21–2.56)	0.037	1.68 (1.26–2.23)	< 0.001
miR-331	1.67 (1.11–2.52)	1.69 (1.12–2.56)	0.097	1.74 (1.29–2.36)	< 0.001
miR-29b	0.66 (0.44–0.99)	0.82 (0.68–0.99)	0.132	0.79 (0.67–0.93)	0.004
miR-1290	0.66 (0.41–1.08)	0.84 (0.72–0.98)	0.097	0.83 (0.73–0.95)	0.006
miR-155	1.57 (1.03–2.38)	1.40 (0.95–2.08)	0.195	1.32 (1.07–1.64)	0.009
miR-148a	0.64 (0.42–0.98)	0.92 (0.7–1.22)	0.511	0.78 (0.62–0.97)	0.023
miR-579	0.57 (0.33–0.97)	0.89 (0.71–1.11)	0.511	0.82 (0.69–0.97)	0.023
miR-1291	0.58 (0.39–0.85)	0.90 (0.73–1.11)	0.511	0.81 (0.68–0.98)	0.027
miR-744*	0.58 (0.38–0.88)	0.89 (0.7–1.13)	0.511	0.82 (0.68–0.99)	0.036
miR-1244	0.64 (0.43–0.95)	0.90 (0.74–1.08)	0.511	0.84 (0.72–0.99)	0.042
miR-486-3p	1.57 (1.02–2.4)	1.12 (0.82–1.53)	0.723	1.28 (1.01–1.63)	0.043
miR-642	1.50 (1.03–2.09)	1.07 (0.88–1.32)	0.723	1.18 (1–1.38)	0.047
miR-340	0.65 (0.44–0.95)	0.93 (0.76–1.15)	0.598	0.87 (0.75–1)	0.057
miR-20a	0.65 (0.43–0.98)	1.02 (0.74–1.41)	0.896	0.87 (0.76–1.01)	0.059
miR-21	0.66 (0.44–0.98)	0.98 (0.76–1.26)	0.723	0.87 (0.74–1.01)	0.067
miR-34a	0.53 (0.31–0.91)	0.95 (0.76–1.2)	0.598	0.85 (0.68–1.05)	0.132
miR-590-3P	0.57 (0.36–0.91)	1.00 (0.87–1.15)	0.896	0.91 (0.81–1.04)	0.165
miR-204	1.57 (1.03–2.38)	0.89 (0.71–1.12)	0.857	1.07 (0.89–1.28)	0.477
miR-493	0.63 (0.39–0.99)	1.16 (0.97–1.39)	0.511	1.03 (0.89–1.2)	0.662
miR-483-5p	0.56 (0.33–0.93)	1.17 (0.98–1.4)	0.481	1 (0.86–1.16)	0.997

Discovery screening based on OR > 1.5 or OR < 0.67. Meta-analysis was conducted based on fixed effect model

*OR* odds ratio, *CI* confidence interval, *FDR* false discovery rate

miR-181a, miR-92a, and miR-424 were the three most significant miRNAs among all 22 miRNAs from the discovery cohort and remained significant in the validation cohort and meta-analysis. Indeed, under the null hypothesis GSEA [17], miR-181a, miR-92a, and miR-424 were significantly overrepresented and enriched among the top six genes on a global miRNA scale (Table 2, Additional file 1: Figure S4).

**Table 2:** Gene set enrichment analysis of 22 candidate miRNAs in whole miRNA set

MicroRNA	Rank in gene list	Enrichment score	Core enrichment
miR-424	0	0.654	Yes
miR-181a	1	0.560	Yes
miR-1291	2	0.545	Yes
miR-744*	3	0.545	Yes
miR-331	5	0.513	Yes
miR-92a	6	0.480	Yes
miR-1244	7	0.446	Yes
miR-486-3p	8	0.383	Yes
miR-493	11	0.328	Yes
miR-204	13	0.323	Yes
miR-34a	18	0.298	Yes
miR-642	21	0.282	Yes
miR-29b	22	0.281	Yes
miR-483-5p	24	0.278	Yes
miR-340	28	0.259	Yes
miR-148a	29	0.257	Yes
miR-590-3P	33	0.253	Yes
miR-1290	47	0.211	No
miR-21	52	0.203	No
miR-579	76	0.151	No
miR-20a	86	0.138	No
miR-155	211	0.051	No

Seventeen of them found to be significantly overrepresented (FDR *q* < 0.001) in ARDS vs at-risk control. miR-181a, miR-92a, and miR-424 are among the top enrich score miRNAs

### Sepsis and pneumonia stratification analysis

Sepsis and pneumonia are the two most common ARDS-predisposing clinical risks and account for the highest percentage of risk in our study cohort. Thus, stratification by sepsis and pneumonia can help validate biomarkers in different risk aspects [21]. According to the risk factor assessment from the validation cohort in Table 3, we selected six miRNAs that showed significant associations with ARDS and performed stratification analyses according to those with sepsis or pneumonia. Among patients with sepsis or pneumonia, miR-424, miR-92a, and miR-181a remained significantly associated with ARDS, reinforcing that the association was independent of sepsis or pneumonia.

**Table 3:** Stratification analysis of miRNA associations with ARDS in validation cohorts

Sepsis			miR-424		miR-92a		miR-181a	
		*N*	OR (95% CI)	*p*	OR (95% CI)	*p*	OR (95% CI)	*p*
	At-risk control	197	Ref.		Ref.		Ref.	
	ARDS	112	0.78 (0.66–0.94)	0.007	1.75 (1.26–2.43)	< 0.001	1.76 (1.21–2.56)	0.003
Pneumonia			miR-424		miR-92a		miR-181a	
		N	OR (95% CI)	*p*	OR (95% CI)	*p*	OR (95% CI)	*p*
	At-risk control	114	Ref.		Ref.		Ref.	
	ARDS	102	0.74 (0.6–0.92)	0.007	1.56 (1.05–2.32)	0.029	1.59 (1.03–2.46)	0.035

Stratify on sepsis or pneumonia only. All models were adjusted for age and gender

*OR* odds ratio, *CI* confidence interval

### miRNA diagnostic performance

Including only ARDS patients from the validation cohort, we performed receiver operating characteristic (ROC) analyses for risk evaluation of ARDS with the LIPS model (AUC = 0.708; 95% CI 0.651–0.766), sepsis (AUC = 0.572; 95% CI 0.573–0.607), and pneumonia (AUC = 0.695; 95% CI 0.651–0.740). AUC values of miR-181a, miR-92a, and miR-424 were larger than that of sepsis, but smaller than AUC values for LIPS or pneumonia. Importantly, specificity and accuracy of the three miRNAs were all larger than those from LIPS, sepsis, or pneumonia (Additional file 1: Table S4), suggesting that these miRNAs have better performance in correctly classifying at-risk controls.

Addition of any one of the three miRNAs significantly increased baseline LIPS AUC, sensitivity, specificity, and accuracy (Table 4). When miR-181a, miR-92a, and miR-424 were computed together with the baseline LIPS model, AUC significantly increased to 0.723 (95% CI 0.667–0.778; *p* = 0.005) (Table 4). Computation of all six miRNAs (miR-181a, miR-92a, miR-424, miR-1290, miR-29b, and miR-331) together with the baseline LIPS model further increased AUC to 0.728 (95% CI 0.674–0.783; *p* = 0.001) (Table 4). These observations suggest that measurement of three miRNAs (miR-181a, miR-92a, and miR-424) from whole blood greatly increased the risk evaluation of ARDS in this population, including AUC, sensitivity, specificity, and accuracy.

**Table 4:** Diagnostic performance of sepsis/pneumonia model and miRNA combined model for ARDS

Combined cohort (*N* = 373)	ARDS vs. at-risk controls							
	AUC (95% CI)	Sensitivity, %	Specificity, %	Accuracy, %	IDI (95% CI)	*p*	Category-free NRI, % (95% CI)	*p*
LIPS model	0.708 (0.651–0.766)	64.35	50.81	56.85	Ref.		Ref.	
miR-181a + LIPS	0.719 (0.661–0.776)	65.03	56.82	59.48	1.36 (0.14–2.58)	0.029	15.24 (−6.38–36.87)	0.649
miR-92a + LIPS	0.716 (0.659–0.773)	64.85	56.73	59.37	1.22 (0.09–2.35)	0.034	8.96 (−12.69–30.61)	0.418
miR-424 + LIPS	0.715 (0.659–0.771)	64.78	56.69	59.32	1.43 (0.12–2.74)	0.033	24.24 (2.71–45.76)	0.237
Extended model 1^a^	0.723 (0.667–0.778)	65.31	56.96	59.67	2.40 (0.72–4.08)	0.005	27.21 (5.72–48.70)	0.014
Extended model 2^b^	0.728 (0.674–0.783)	65.70	57.14	59.92	3.18 (1.28–5.09)	0.001	36.93 (15.58–58.28)	<0.001

*AUC* area under the curve, *IDI* integrated discrimination improvement, *NRI* net reclassification index, *LIPS* Lung Injury Prediction Score

^a^Extend model 1: LIPS + miR-181a + miR-92a + miR-424

^b^Extend model 2: LIPS + miR-181a + miR-92a + miR-424 + miR-1290 + miR-29b + miR-331

Relative IDI values for miR-181a, miR92a, and miR-424 were 1.36 (95% CI 0.14–2.58; *p* = 0.029), 1.22 (95% CI 0.09–2.35; *p* = 0.034), and 1.43 (95% CI 0.12–2.74; *p* = 0.033), respectively. Of note, combination of miR-181a, miR-92a, and miR-424 increased IDI to 2.40 (95% CI 0.72–4.08; *p* = 0.005) and category-free NRI to 27.21% (95% CI 5.72–48.70; *p* = 0.014) (Table 4).

## Discussion

ARDS is a life-threatening inflammatory disease of the lung. Although a mechanical ventilation strategy has been shown to influence mortality in this syndrome, there is currently no proven pharmacologic treatment despite more than 30 completed or ongoing clinical trials. The mortality rate of ARDS remains high [1, 22, 23]; therefore, early diagnosis and prevention are essential. The LIPS model has been used to detect potential risk factors for ARDS, using clinical predisposing conditions based on clinical observations. While this method is appropriately sensitive, it may not accurately reflect the pathophysiological process of ARDS [18].

To our knowledge, this study is the first to use whole blood samples from a large population of ARDS patients and critically ill, at-risk controls and to use a discovery and independent validation cohort study design with rigorous statistical analysis of a high-throughput miRNA set. The primary selected 22 miRNAs from the discovery cohort were further validated in an independent cohort and with subsequent meta-analysis. Such diligent analyses may offer an advantage for miRNA profiling and greatly reduce risk of misrepresentation from miRNA expression noise that typically results from additional serum or plasma isolation steps and sample contamination. Although patient collection spanned 10 years, we carefully selected high-quality samples and controlled all experiments by grouping patients into a randomly selected discovery cohort and a validation cohort according to dates of sample collection. We used identical amounts of RNA input in all experiments to control against bias caused by variation due to different RNA input amounts. We also distributed samples such that age, sex, case-control status, and RNA quality were balanced with respect to the day of purification and the day of analysis or plate number and randomized within each day and plate to reduce confounding factors from technical variation, such as plate-to-plate variation and purification differences.

miRNAs have been used successfully as biomarkers for chronic diseases, such as pancreatic and gastric cancers [9, 24]. This study identified three promising miRNAs—miR-181a, miR-92a, and miR-424—that are associated with human ARDS. GSEA confirmed that they were significantly overrepresented in ARDS cases compared to controls in these study cohorts, although there is no evidence directly linking these miRNAs to ARDS. However, multiple recent studies provide indirect evidence for the miRNAs’ involvement in dysregulated ARDS signaling pathways [25–27].

Here, we report that miR-181a and miR-92a are associated with ARDS risk in all tested cohorts and meta-analysis. These findings are consistent with prior studies in inflammation and endothelial cell injury, which are common in ARDS [28]. miR-181a is a key regulator of T-cell development and T-cell receptor signaling threshold [25]. Increased miR-181a expression in mature T-cells augments cell sensitivity to peptide antigens. Further, T-cell responses decline with age due to an age-associated defect in T-cell receptor signaling, which is caused by increased expression of phosphatase 6 and miR-181a. The miR-181 family is also upregulated in asthma airway inflammation [29] and neutrophil regulation [30], which both play crucial roles in the pathophysiology of ARDS [2].

miR-92a inhibits endothelial cell angiogenesis and impairs endothelial cell function [26, 31–33]. miR-92a also targets Krüppel-like factor 2 (KLF2), KLF4, and sirtuin 1, thereby promoting inflammatory responses [26, 33, 34]. Further, lung microvascular endothelium injury-associated pulmonary edema is a hallmark of ARDS [35, 36]. When miR-92a is overexpressed, blood vessel growth and functional recovery of damaged tissue are restricted [31], which may enhance the incidence of pulmonary edema and ARDS.

In contrast, miR-424 was a protective factor for ARDS in our study (Table 1). miR-424 is downregulated in pulmonary artery hypertension (PAH) via apelin and fibroblast growth factor 2 signaling in pulmonary artery endothelial cells [27]. PAH is commonly observed in ARDS patients, who can have hypoxemia that promotes pulmonary vasoconstriction to give rise to PAH. Hypoxia-induced miR-424 plays an important role in vascular remodeling and angiogenesis in endothelial cells [37]. Low oxygen levels affect cells and tissues during wound healing as well as during pathological conditions, such as stroke. As a consequence, miR-424 signaling is activated in endothelial cells to stabilize hypoxia-inducible factors [37]. These prior studies support our finding that miR-424 expression may exert a protective effect against ARDS.

Of note, sepsis and pneumonia had lower prevalence in at-risk controls than ARDS subjects in this population. Thus, we conducted stratification analysis to prove that the top three miRNAs (miR-181a, miR-92a, and miR-424) remained significantly associated with ARDS regardless of sepsis and pneumonia imbalance between our two cohorts. LIPS is currently considered a standard clinical prediction model and is associated with ARDS risk and complications [18]. A recent study evaluating LIPS on ARDS development showed LIPS has an AUC of 0.70 [38], which is consistent with our results [38]. All three miRNAs selected from our two cohorts had similar specificity and accuracy in predicting ARDS to that of LIPS. Further, incorporation of these miRNAs with LIPS further increased the potency and accuracy of ARDS risk estimate. Therefore, miRNAs identified from this study may have incremental utility to that of LIPS for future ARDS risk evaluation.

This study focused mainly on three miRNAs selected from the validation cohort. However, that does not mean that the remaining 19 miRNAs identified in the discovery cohort are irrelevant. Some of these miRNAs have been implicated in inflammatory signaling pathways and may also be ARDS candidate risk factors. For example, miR-155 and miR-21 are functionally related and contribute to NF-κB signaling [39], an important pathway for innate and adaptive immunity and inflammation. miR-155 is also upregulated in asthma, and the miR-29 family is upregulated in adult lungs. Further, miR-21 has been shown to play multiple roles in different pulmonary diseases, such as idiopathic pulmonary fibrosis and pulmonary arterial hypertension, by targeting several immune receptors and cytokines, including IL-12 and SMAD7 [29]. Further investigation is needed to inform the involvement of these miRNAs in ARDS, which might provide a better understanding of mechanisms underlying the disease.

The miRNAs identified for ARDS risk were different from the ones we identified in previous prior study [11]. ARDS patients have a rapid change in the syndrome progress, from the initial pulmonary tissue damage and inflammation/infection to later wherein some of them developed MOF. And miRNAs are functional. This fact explains why miRNAs signal can be different under various disease biological conditions even with the same phenotype. The miRNAs we found in the other study were mainly associated with organ failure, whereas the miRNAs in current study were mainly associated with endothelial cell damage and inflammatory response.

However, we also acknowledge limitations of our study. First of all, our results were only based on 294 (out of 754) miRNAs that passed stringent QC criteria. It is possible that miRNAs did not pass QC are functionally related to ARDS. More studies, such as miRNA injection in ARDS mouse model, are needed to proof their functionality. The diagnostic power of our miRNAs might not be sufficiently strong. However, unlike other similar studies [8, 24], our control subjects were at-risk patients who share more features with ARDS cases, which may have reduced confounding variables compared to the use of healthy controls. In addition, ARDS is considered a complicated syndrome with multiple etiologies, so a single or a few miRNAs might not show strong signals for all ARDS patients. This concept was recently confirmed in an ARDS randomized clinical trial, which concluded that aspirin has no beneficial effect for ARDS prevention [40]. Aspirin has direct effect only on platelet function-related mechanistic pathways [41]. Although alteration in platelet function was found during ARDS development [42], it was not the only mechanistic pathway. Also, our study was based on a single geographic region—a geographically different, external cohort in a similar study setting would be helpful to further validate our findings. In this study, miRNA target gene expression data are not available. Future research on such targets will be informative for validating the functions of the identified miRNA in ARDS and provide more comprehensive understanding of mechanistic knowledge.

## Conclusions

This study links whole blood expression of miR-181a, miR-92a, and miR-424 to ARDS. Inflammatory response markers miR-181a and miR-92a were significantly elevated in ARDS patients, while pulmonary artery endothelial cell anti-inflammation marker miR-424 was significantly reduced in ARDS patients. Further, expression patterns of our miRNA biomarkers may provide an in-depth molecular understanding of ARDS among at-risk patients beyond clinical factors, such as sepsis and pneumonia. In addition, combining these miRNA biomarkers with the LIPS model may further improve ARDS diagnosis.

## Additional file

Additional file1: Table S1. Study required risk factors for ARDS on admission to ICU [6]. Table S2. Demographic characteristics of MEARDS miRNA study cohorts (*n* = 529). Table S3. MicroRNA candidate screening in discovery study. Table S4. Diagnostic performance of sepsis, pneumonia, and miRNA biomarkers for ARDS. Figure S1. MEARDS cohort recruitment process. Figure S2. Sample A (A) and sample B (B) from two patients both showed strong correlations between duplicate samples on different chips and different profiling day in discovery study (*R* ^2^ = 0.99), indicating that detectable miRNAs (after meeting quality control criteria) are experimentally consistent. Figure S3. Sample duplicate consistency between discovery and validation phase (after meeting quality control criteria). The figure showed high correlation (*R* ^2^ = 0.90) between miRNA expression in discovery study and validation study. Figure S4. Gene set enrichment analysis of 22 candidate miRNAs. Seventeen of them found to be significantly overrepresented (FDR *q* < 0.001) in ARDS vs at-risk control. miR-181a, miR-92a, and miR-424 are among the top enrich score miRNAs. Figure S5. Post hoc power calculation of logistic regression was calculated using G power (3.1.9). Under the null hypothesis, we assume the odds ratio equals to 1.5 with total sample size of 156; thus, we have a power (1 − *β* error probability) of 0.79 to detect differentially expressed miRNAs. (DOCX 125 kb)
