# Supplementary material for: Whole blood microRNA markers are associated with acute respiratory distress syndrome
Source: Intensive Care Med Exp. 2017 Aug 30;5:38. doi: 10.1186/s40635-017-0155-0 (PMC5577350; doi:10.1186/s40635-017-0155-0)
Supplement: Additional file1: Table S1. — Study required risk factors for ARDS on admission to ICU [6]. Table S2. Demographic characteristics of MEARDS miRNA study cohorts (n = 529). Table S3. MicroRNA candidate screening in discovery study. Table S4. Diagnostic performance of sepsis, pneumonia, and miRNA biomarkers for ARDS. Figure S1. MEARDS cohort recruitment process. Figure S2. Sample A (A) and sample B (B) from two patients both showed strong correlations between duplicate samples on different chips and different profiling day in discovery study (R 2 = 0.99), indicating that detectable miRNAs (after meeting quality control criteria) are experimentally consistent. Figure S3. Sample duplicate consistency between discovery and validation phase (after meeting quality control criteria). The figure showed high correlation (R 2 = 0.90) between miRNA expression in discovery study and validation study. Figure S4. Gene set enrichment analysis of 22 candidate miRNAs. Seventeen of them found to be significantly overrepresented (FDR q < 0.001) in ARDS vs at-risk control. miR-181a, miR-92a, and miR-424 are among the top enrich score miRNAs. Figure S5. Post hoc power calculation of logistic regression was calculated using G power (3.1.9). Under the null hypothesis, we assume the odds ratio equals to 1.5 with total sample size of 156; thus, we have a power (1 − β error probability) of 0.79 to detect differentially expressed miRNAs. (DOCX 125 kb) [file 40635_2017_155_MOESM1_ESM.docx]

**Supplemental Digital Content**

**Whole Blood MicroRNA Markers are Associated with Acute Respiratory Distress Syndrome**

Zhaozhong Zhu, Liming Liang, Ruyang Zhang, Yongyue Wei, Li Su, Paula Tejera, Yichen Guo, Zhaoxi Wang, Quan Lu, Andrea A. Baccarelli, Xi Zhu, Ednan K. Bajwa, B. Taylor Thompson, Guo-Ping Shi, David C. Christiani

**Supplemental Methods and Results**

**Patient selection**

As part of the MEARDS (molecular epidemiology study of ARDS, Clinical Trail #: NCT00006496), admissions to the neurologic, cardiac, medical and surgical intensive care units (ICUs) of the Massachusetts General Hospital (MGH, Boston, MA) and Beth Israel Deaconess Medical Center (BIDMC, Boston, MA) were screened daily for risk factors for ARDS (Table S1). All ARDS subjects met with Berlin definition: the timing of ARDS should be within 1 week of a known clinical insult or new or worsening respiratory symptoms; the chest imaging should show bilateral opacities (not fully explained by effusions, lobar/lung collapse, or nodules); the respiratory failure not fully explained by cardiac failure or fluid overload; ARDS severity is based on PaO2/FiO2 ratio [[1](#_ENREF_1)].

**Study subjects**

Between 2005 and 2014, we collected 530 whole blood samples from MEARDS. Of the 530 patients, 156 patients were allocated to the discovery cohort, 373 patients were allocated to the validation cohort [[2](#_ENREF_2)]. One sample was excluded due to few detectable miRNAs in validation cohort.

**cDNA synthesis and microRNA (miRNA) profiling**

For each sample, we used 200 ng total RNA containing the small RNA fraction for reverse transcription with Megaplex™. The small RNA fraction was not enriched to avoid loss of longer control transcripts (snoRNAs). Each miRNA plate contained 2 TaqMan® MicroRNA assay endogenous housekeeping gene controls (RNU44, RNU48) to aid in data normalization.

**Cohorts and experiment data consistency**

In the discovery cohort, we included samples from two patients to confirm data consistency between assays from different experiments. Each OpenArray plate contained two samples and the system can run four plates simultaneously as one experiment. Correlation between the two independent experiments was high (R^2^=0.99) (Supplementary Figure S2). In addition, we also used one patient sample to examine data consistency between the discover cohort and validation cohort, which used a different assay platform from that of OpenArray plate (Supplementary Figure S3). The validation cohort used customized OpenArray TaqMan MicroRNA assay system, which shared the same platform of QuantStudio™ 12K Flex Real-Time PCR System. Each sample was tested in triplicate to lower the effect of technical bias. The benefit of using the same platform in both validation cohorts was to reduce the risk of platform inconsistencies between the validation cohort and discovery cohort (Supplementary Figure S3). We used identical amounts of RNA input and endogenous housekeeping genes RNU44, RNU48 for primary data normalization. In order to minimize the risk of losing true signals due to one housekeeping normalization, we also used global or quantile normalization or raw Ct value without normalization to screen miRNA with large fold change.[[2](#_ENREF_2)] Total RNA (100 ng/each) containing the small RNA fraction was used per reverse transcription (RT) reaction (both pool A and pool B). Total RNA (100 ng) was added in 3 μL to each well containing RT reaction mix (3 μL of water for the no-template control reactions). The cDNA was run through 40 cycles of 16 ^o^C for 2 minutes, 42 ^o^C for 1 minute, and 50 ^o^C for 1 second. Reactions were then held at 85 ^o^C for 5 minutes and cooled to 4 ^o^C for storage.

**miRNA data quality control and normalization**

For all quality control (QC) procedures, we used the following criteria to identify reliable miRNAs: amplification score >1.1, Cq confidence >0.8, high expression (Ct<30), and missing percent less than half of samples.[[3](#_ENREF_3)] Four normalization methods––endogenous gene, global normalization, quantile normalization, and keep as raw—were applied using R/Bioconductor, for further statistical analysis, where endogenous gene normalization (average of RNU44 and RNU48 expression) was assigned as primary. Imputation was used to handle missing miRNA data (Supplement).

**Missing data and Imputation**

We applied a strict quality control (QC) procedure in order to assure the quality of results. After QC, we only used miRNAs with missing percent less than half of samples because imputation should be conducted based on some amount of original data. If the missing percent is more than half, the imputation methods should not be implemented due to low accuracy.[[3](#_ENREF_3)] We assumed that the Ct values were not missing at random but missing due to low expression of miRNA in the sample. Therefore, we imputed missing data using the 95th percentile of the same miRNA for cases or controls.[[2](#_ENREF_2)]

**Integrated discrimination improvement (IDI) and net reclassification improvement (NRI)**

IDI and NRI are two statistics proposed to evaluate the significance of novel predictors [[4](#_ENREF_4)]. The IDI measures the new model’s improvement in average sensitivity without sacrificing average specificity. The relative IDI is defined using Equation 1 [[5](#_ENREF_5)]. P is the predicted probability for each subject, derived from logistic regression model. The NRI measures the correctness of reclassification of subjects based on their predicted probabilities of events using the new model. The category-free NRI is defined using Equation 2 [[5](#_ENREF_5)].

$$IDI_{relative}=\frac{{(\bar{P}}_{case}^{model2}-\bar{P}_{case}^{model1})+{(\bar{P}}_{control}^{model1}-\bar{P}_{control}^{model2})}{\bar{P}_{case}^{model1}-\bar{P}_{control}^{model1}} (1)$$

$$NRI_{category-free}=\frac{N_{P^{model2}>P^{model1}-}N_{P^{model2}<P^{model1}}}{N_{case}}+\frac{N_{P^{model2}<P^{model1}-}N_{P^{model2}>P^{model1}}}{N_{control}} (2)$$

The N_case_ and N_control_ are the number of cases and controls, respectively. P is the predicted probability for each subject, derived from logistic regression model.

**Supplemental Tables and Figures**

**Supplementary Table S1.** Study required risk factors for ARDS on admission to ICU [[6](#_ENREF_6)]

| **Sepsis syndrome:** As defined by SCCM to be a known or suspected source of systemic infection and at least two of the following: a) temperature >38 ^o^C or <36 ^o^C; b) heart rate >90 beats/min; c) respiratory rate >20 breaths/min or Pa_CO2_<32 mm Hg; d) WBC> 12,000/mm^3^, <4000/mm^3^, or >10% bands. |
| --- |
| **Septic shock:** Fulfill requirements for sepsis and one of the following: a) SBP <90 mm Hg or  reduction of ≥40 mm Hg from baseline for ≥30 mins unresponsive to 500 mL of fluid  resuscitation; b) need for vasopressors to maintain SBP 90 mm Hg or within 40 mm Hg of baseline. |
| **Pneumonia:** Fulfill two or more of the following: a) new infiltrate on CXR; b) temperature >38.3°C or <36.0°C or WBC >12,000 or <4000 or >10% bandemia; c) positive microbiologic culture. |
| **Trauma:** Defined as multiple fractures and/or pulmonary contusions. Multiple fractures are defined as a fracture of two long bones, an unstable pelvic fracture, or one long bone and a pelvic fracture. Pulmonary contusion is defined as infiltrates on CXR within 8 hrs of admission to the emergency room and evidence of blunt trauma to the chest such as fractured ribs or ecchymosis overlying the infiltrate. |
| **Multiple transfusions:** Defined as receiving ≥8 units of PRBCs within 24 hrs. |
| **Aspiration:** Defined as witnessed or documented aspiration event or the retrieval of gastric contents from the oropharynx, endotracheal tube, or bronchial tree |

Abbreviations: SCCM, Society of Critical Care Medicine; WBC, white blood cell count; SBP, systolic blood pressure; CXR, chest radiograph; PRBC, packed red blood cells.

**Supplementary Table S2.** Demographic characteristics of MEARDS miRNA study cohorts (n=529).

|  | Discovery cohort | | Validation cohort | |
| --- | --- | --- | --- | --- |
|  | (n=156) | | (n=373) | |
|  | ARDS cases | ARDS controls | ARDS cases | ARDS controls |
| ***N*** | 78 | 78 | 121 | 252 |
| Age, median years (range) | 64.0 (22-87) | 61.5 (22-90) | 53.0 (18-94) | 62 (18-101) |
| Female, n (%) | 17 (21.79) | 17 (21.79) | 51 (42.15) | 99 (39.29) |
| **Baseline severity of illness (1st 24 hours of ICU admission)** | |  |  |  |
| APACHE III, median (range)* | 85 (26-135) | 64 (19-123) | 65 (14-142) | 63 (11-130) |
| Systolic BP, <90 mmHg, n (%) | 53 (67.95) | 50 (64.12) | 85 (70.25) | 161 (63.89) |
| Heart rate, >100 beats/min, n (%) | 58 (74.36) | 45 (57.69) | 96 (79.34) | 163 (64.68) |
| Respiratory rate, >30 breaths/min, n (%) | 41 (52.56) | 23 (29.49) | 53 (43.80) | 59 (23.41) |
| Acidosis (pH < 7.35) | 39 (50) | 34 (43.59) | 47 (38.84) | 82 (32.54) |
| Hypoalbuminemia (<35) | 46 (58.97) | 42 (53.85) | 71 (58.68) | 137 (54.37) |
|  |  |  |  |  |
| **Comorbidities** |  |  |  |  |
| Diabetes, n (%) | 18 (24.00) | 24 (30.77) | 27 (22.31) | 71 (28.17) |
|  |  |  |  |  |
| **Predisposing conditions for ARDS** |  |  |  |  |
| Sepsis syndrome, n (%) | 71 (91.03) | 62 (79.49) | 112 (92.56) | 197 (78.17) |
| Septic shock, n(%) | 62 (79.49) | 46 (58.97) | 94 (77.69) | 146 (57.94) |
| Pneumonia, n (%) | 66 (84.62) | 41 (52.56) | 102 (84.30) | 114 (45.24) |
| Trauma, n (%) | 0 (0) | 1 (1.28) | 2 (1.65) | 18 (7.14) |
| Multiple transfusions, n (%) | 3 (3.85) | 7 (8.97) | 7 (5.79) | 29 (11.51) |
| Lung contusion, n (%) | 1 (1.28) | 4 (5.13) | 7 (5.79) | 13 (5.16) |
| Aspiration, n (%) | 7 (8.97) | 4 (5.13) | 12 (9.92) | 12 (4.76) |

*Abbreviation: APACHE III, Acute Physiology and Chronic Health Evaluation III.

**Supplementary Table S3.** MicroRNA candidate screening in discovery study.

| Discovery study microRNA screening | |
| --- | --- |
| MicroRNA | OR (95% CI)^ |
| miR-181a | 1.75 (1.03-2.97) |
| miR-331 | 1.67 (1.11-2.52) |
| miR-92a | 1.6 (1.11-2.31) |
| miR-204 | 1.57 (1.03-2.38) |
| miR-486-3p | 1.57 (1.02-2.4) |
| miR-155 | 1.57 (1.03-2.38) |
| miR-642 | 1.50 (1.03-2.09) |
| miR-1290 | 0.66 (0.41-1.08) |
| miR-21 | 0.66 (0.44-0.98) |
| miR-29b | 0.66 (0.44-0.99) |
| miR-340 | 0.65 (0.44-0.95) |
| miR-20a | 0.65 (0.43-0.98) |
| miR-1244 | 0.64 (0.43-0.95) |
| miR-148a | 0.64 (0.42-0.98) |
| miR-493 | 0.63 (0.39-0.99) |
| miR-1291 | 0.58 (0.39-0.85) |
| miR-744* | 0.58 (0.38-0.88) |
| miR-579 | 0.57 (0.33-0.97) |
| miR-590-3P | 0.57 (0.36-0.91) |
| miR-483-5p | 0.56 (0.33-0.93) |
| miR-34a | 0.53 (0.31-0.91) |
| miR-424 | 0.52 (0.29-0.93) |

Abbreviation: OR, Odds ratio.

^Logistic regression model adjusted for sepsis and pneumonia; Odds ratio is fold change in the context of gene expression study. Risk factor is fold change greater than 1.50, protecting factor is fold change less than 0.67 (1/1.5=0.67).

**Supplementary Table S4.** Diagnostic performance of sepsis, pneumonia, and miRNA biomarkers for ARDS.

|  | ARDS *vs.* at-risk controls | | | |
| --- | --- | --- | --- | --- |
| Combined cohort (N=373) | AUC (95% CI) | Sensitivity % | Specificity % | Accuracy % |
| LIPS model | 0.708 (0.651-0.766) | 64.35 | 50.81 | 56.85 |
| Sepsis | 0.572 (0.537-0.607) | 55.00 | 9.04 | 37.55 |
| Pneumonia | 0.695 (0.651-0.740) | 63.35 | 15.86 | 41.68 |
| miR-181a | 0.590 (0.529-0.652) | 56.37 | 52.63 | 53.86 |
| miR-424 | 0.586 (0.524-0.648) | 56.10 | 52.33 | 53.61 |
| miR-92a | 0.583 (0.523-0.644) | 55.90 | 52.41 | 53.54 |

Abbreviation: LIPS, lung injury prediction score; AUC, area under the curve.

**Supplementary Figure S1.** MEARDS cohort recruitment process.

Yes

Yes

Yes

Yes

Admission to adult ICUs of MGH & BIDMC

Risk factors for ARDS (sepsis, septic shock, pneumonia, etc.)

Did not develop ARDS?

Developed ARDS?

Meets any exclusion criteria?

Informed consent

Not eligible

ARDS Case

Not eligible

At-risk control

No

No

Yes

Yes

No

**A**

**B**

**Supplementary Figure S2.** Sample A (**A**) and sample B (**B**) from two patients both showed strong correlations between duplicate samples on different chips and different profiling day in discovery study (R^2^=0.99), indicating that detectable miRNAs (after meeting quality control criteria) are experimentally consistent.

**Supplementary Figure S3.** Sample duplicate consistency between discovery and validation phase (after meeting quality control criteria). The figure showed high correlation (R^2^=0.90) between miRNA expression in discovery study and validation study.


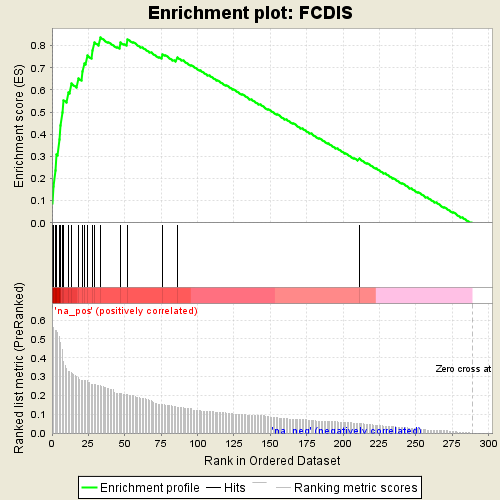


**Supplementary Figure S4.** Gene set enrichment analysis of 22 candidate miRNAs. 17 of them found to be significantly overrepresented (FDR q < 0.001) in ARDS vs at-risk control. miR-181a, miR-92a, and miR-424 are among the top enrich score miRNAs.

**Supplementary Figure S5.** Post hoc power calculation of logistic regression was calculated using G power (3.1.9). Under the null hypothesis, we assume the odds ratio equals to 1.5 with total sample size of 156, thus we have a power (1-β error probability) of 0.79 to detect differentially expressed miRNAs.

**References**

1. Force ADT, Ranieri VM, Rubenfeld GD, Thompson BT, Ferguson ND, Caldwell E, Fan E, Camporota L, Slutsky AS, (2012) Acute respiratory distress syndrome: the Berlin Definition. JAMA : the journal of the American Medical Association 307: 2526-2533

2. Schultz NA, Dehlendorff C, Jensen BV, Bjerregaard JK, Nielsen KR, Bojesen SE, Calatayud D, Nielsen SE, Yilmaz M, Hollander NH, Andersen KK, Johansen JS, (2014) MicroRNA biomarkers in whole blood for detection of pancreatic cancer. JAMA : the journal of the American Medical Association 311: 392-404

3. Thermo Fisher Co. (2015) Taqman MicroRNA® Assays FAQs. In: Editor (ed)^(eds) Book Taqman MicroRNA® Assays FAQs. City, pp.

4. Pencina MJ, D'Agostino RB, Sr., D'Agostino RB, Jr., Vasan RS, (2008) Evaluating the added predictive ability of a new marker: from area under the ROC curve to reclassification and beyond. Statistics in medicine 27: 157-172; discussion 207-112

5. Zhang R, Chu M, Zhao Y, Wu C, Guo H, Shi Y, Dai J, Wei Y, Jin G, Ma H, Dong J, Yi H, Bai J, Gong J, Sun C, Zhu M, Wu T, Hu Z, Lin D, Shen H, Chen F, (2014) A genome-wide gene-environment interaction analysis for tobacco smoke and lung cancer susceptibility. Carcinogenesis 35: 1528-1535

6. Gong MN, Thompson BT, Williams P, Pothier L, Boyce PD, Christiani DC, (2005) Clinical predictors of and mortality in acute respiratory distress syndrome: potential role of red cell transfusion. Critical care medicine 33: 1191-1198
